# Livestock guinea pigs in Ecuador as reservoirs of zoonotic protozoa and helminths

**DOI:** 10.3389/fvets.2025.1658485

**Published:** 2025-09-24

**Authors:** Mauricio Xavier Salas-Rueda, Froilan Patricio Garnica-Marquina, Verónica Patricia Curipoma-Maisincho, Katherine Natalia Chávez Toledo, Erika Carolina Rocano-Marcatoma, Solon Alberto Orlando, Fabricio Arcos Alcivar, Angel Sebastian Rodriguez-Pazmiño, Javier Hermoso de Mendoza, Miguel Angel Garcia-Bereguiain

**Affiliations:** ^1^GLOBALGEN, Universidad Politécnica Salesiana, Cuenca, Ecuador; ^2^Universidad Católica Santiago de Guayaquil, Guayaquil, Ecuador; ^3^Instituto Nacional de Investigación en Salud Pública, Guayaquil, Ecuador; ^4^Universidad Espíritu Santo, Guayaquil, Ecuador; ^5^Universidad Ecotec, Guayaquil, Ecuador; ^6^One Health Research Group, Universidad de Las Américas, Quito, Ecuador; ^7^Departamento de Patología Infecciosa, Facultad de Veterinaria, Universidad de Extremadura, Badajoz, Spain

**Keywords:** guinea pigs, livestock, Ecuador, parasites, helminths, protozoans, *Giardia*, *Fasciola hepatica*

## Abstract

Guinea pigs *(Cavia porcellus)* are bred as laboratory animal models and pets worldwide. However, they are also raised as livestock in South American countries from the Andean region, including Ecuador. Despite their importance for the rural local economy, no specific management guidelines for guinea pig farming have been developed by Ecuadorian animal or public health authorities. Moreover, several reports have shown the carriage of diverse zoonotic pathogens in guinea pigs. In this study, the prevalence of enteric protozoan and helminths in guinea pigs from Ecuador was analyzed. Fecal samples from 765 guinea pigs from 153 farms were collected. The overall prevalence of parasitism was 86.0% (95% CI: 83.4–88.3). Five different genera of protozoan parasites, which include zoonotic species (*Giardia, Cryptosporidium, Entamoeba, Eimeria*, and *Balantidium*), were found, and the overall prevalence of protozoans was 56.21% (95% CI: 52.7–59.7). Seven different genera of helminth parasites, including zoonotic species *Capillaria, Fasciola, Trichostrongylus*, and *Trichuris*, were identified, with an overall helminth prevalence of 70.1% (95% CI: 66.8–73.2). Several risk factors related to animal production practices were considered and the multivariate analysis identified that forage based feeding, the use of wire cages and interaction with other domestic animals were associated with higher prevalence of parasitism. Our results highlight the role of guinea pigs as a reservoir for zoonotic enteric parasites of public health relevance in Ecuador. Moreover, our study is the first report of *Fasciola hepatica* in Ecuadorian guinea pig. Animal and public health guidelines from a One Health perspective must be implemented to prevent occupational exposure to parasites in guinea pig farming and to ensure food security in the Andean region, where this animal is a significant source of protein in the human diet.

## 1 Introduction

The guinea pig *(Cavia porcellus)* is bred worldwide either as a laboratory model or a pet (1). Native to the Andean region of the Americas, guinea pigs are raised for ceremonial events and livestock in Colombia, Ecuador, Peru, and Bolivia (2). Guinea pig farming modalities vary from backyard breeding in groups of a few animals per household for family consumption to larger establishments with thousands of animals for commercial purposes (3). Only in Ecuador, at least 710,000 families are involved in guinea pig farming, with an estimated annual production of 50 million animals that are destined for sale and family consumption (4). Moreover, guinea pig breeding as livestock has been recently introduced in some African countries, such as Cameroon and Benin (5, 6). Despite its importance to the local rural economy, breeding is still performed mainly in a traditional manner. Lack of veterinary counseling or animal health-specific guidelines are usual, compromising food safety (3, 4).

The status of guinea pigs as zoonotic reservoirs is well known, given their frequent use for experimental infections as laboratory animal models, as well as pets (3). However, this knowledge remains very limited for guinea pigs raised as livestock (3). Several zoonotic pathogens have already been identified in livestock guinea pigs, including respiratory pathogens such as influenza virus, antibiotic-resistant *Staphylococcus aureus* and *Streptococcus pneumoniae*, as well as yeasts (7–10). Also enteric pathogens like *Campylobacter jejuni* (11), and *Toxoplasma gondii* have been described (12, 13). Additionally, several studies have shown the role of guinea pigs as zoonotic reservoirs for enteric parasites (6, 14–17). Most of these studies were conducted in Ecuador and Peru, reporting a high prevalence of protozoan parasites, including *Giardia, Blastocystis, Entamoeba, Eimeria*, and *Cryptosporidium*, as well as several species of helminths (14–17). Moreover, a study carried out in Cameroon has reported a high prevalence of *Giardia, Cryptosporidium*, and helminths in livestock guinea pigs (6), as well.

Enteric parasites, including helminths and protozoans, are significant contributors to the global burden of disease, particularly in rural, low-income settings in tropical regions such as Ecuador (18). In these endemic areas, chronic parasitic infections during childhood have a strong link with stunting, the most common form of malnutrition in children, affecting physical growth and cognitive impairment (18). Moreover, the protozoan parasites *Cryptosporidium* spp., *Giardia duodenalis*, and *Entamoeba histolytica* are relevant diarrhea-causing pathogens globally, transmitted fecally either directly through contact with infected humans and other animals or indirectly via the ingestion of contaminated food or water (6, 18). Although the carriage of these parasites has already been reported in guinea pigs, the role of guinea pigs as a reservoir for enteric pathogens is still poorly understood.

Despite the importance of guinea pig farming for rural communities in the Andean Region, there is an important gap of knowledge related to infectious diseases affecting guinea pig production and public health. Thus, we aim to study the prevalence of protozoan and helminth parasites in livestock guinea pigs from Ecuador and to identify the potential risk factors related to animal production practices on this species.

## 2 Materials and methods

### 2.1 Study setting and sample collection

Guinea pigs from farms and households located in Paute canton in Azuay province of Ecuador were included in the study. This province is located in the Andean region, at an elevation of 2,500 m above sea level, and it is one of the leading producers of guinea pig meat in the country (9). Samples were collected from 153 guinea pig farms. These farms were divided into different categories related to the characteristics of animal production features (see Supplementary Table 1):

1) Three types according to the number of animals per farm: backyard production (< 100; this backyard production includes a traditional guinea pig breeding practice among indigenous communities in Azuay province where the animals roam freely within the kitchen area of the household), small scale (101–500) and large scale (>500).
2) Two types of farms were considered depending of the type of cage: “jaula” and “poza”. These two categories correspond to the main housing systems used in guinea pig farming. A “jaula” is a suspended wire cage, elevated from the ground, usually built with metal mesh or wooden frames, which facilitates ventilation and cleaning by preventing direct contact between animals and their waste. This system is considered more hygienic. In contrast, a “poza” is a ground-level enclosure, generally constructed with wooden, brick, or adobe walls on a dirt or cement floor. “Pozas” are designed to house larger groups of guinea pigs together and represent the most traditional and widespread system in rural households due to their low cost and ease of construction. However, they demand careful management of hygiene, humidity and feeding practices.
3) Two categories of farms were stablished according to the type of feeding: fresh forage and mixed fresh forage/balanced feed.
4) Two categories were defined depending on the presence or absence on other animals on the farms such as dogs, cats, poultry or other backyard animals.
5) Two categories were considered depending of the presence/absence of veterinarian care in the farm.

Samples were collected from October 2020 to February 2021. Because of the lack of an official census of guinea pig farms in Ecuador, the inclusion of farms in this study was done at convenience following a “snowball” approach to contact and recruit neighbor guinea pig farmers within Paute canton in Azuay province. The total number of 153 farms included 30 backyard production farms, 116 small-scale farms, and seven large-scale farms. Of those farms, 102 had breeding in “poza” and 51 had breeding in “jaula”. Twenty-four were raised exclusively on fresh forage, and 129 were fed with fresh forage supplemented with balanced feed. Twenty six farms had the presence of other animal species, whereas 129 farms maintained no interspecies contact. Only 11 operations had veterinary advisory services, whereas 142 farms lacked specialized support.

Five fecal samples from 5 guinea pigs located in different cages were collected on each farm, meaning a total number of 765 guinea pigs included in the study. A rectal swab was collected for each animal. Samples were stored at 4 °C until they arrived at the laboratory for analysis.

### 2.2 Detection of gastrointestinal parasites in fecal samples

All the fecal samples collected from guinea pigs were examined within 24 h after collection. Only one lab tech with previous expertise in parasite identification performed microscopic analysis. Two approaches were used for the detection of cysts and oocysts of the protozoa, and eggs and larvae of the helminths: direct smear and formal-ether concentration (14, 19). For the direct smear, the flotation method was used by mixing a saturated salt solution (NaCl) with ~2 mg of feces on a microscopy slide and examined for helminth eggs; a drop of iodine was mixed with ~2 mg of feces for protozoan cysts examination. For the formal-ether concentration, 50 mg of fecal material was thoroughly mixed with 2 ml of 10% formalin, and then was filtered through a fecal parasite strainer into an empty tube. The filtrate was mixed with 1.5 mL of ether. This mixture was then shaken vigorously for 1 min and centrifuged at 500 × g for 2 min. An unstained wet mount of the sediment was used for the detection of helminth eggs and larvae. For protozoan cysts, a thin, iodine-stained wet mount of the sediment was used. Both direct smear and formal-ether concentration samples were analyzed with 10 × and 40 × lens. Parasites were identified in this study using established morphological criteria. Eggs, cysts, and oocysts were identified based on size, shape, color, shell thickness, and the presence of characteristic internal structures, following the morphological keys previously described for helminths (20, 21) and protozoa (22).

### 2.3 Statistical analysis

Data management and descriptive, univariate and multivariate (logistic regression) analysis were performed using EPIINFO 7.2.5.0, and statistical significance was set at 0.05. The prevalence values were calculated with 95% confidence intervals.

### 2.4 Ethics statement

The study was done according to national regulation in Ecuador. In this sense, as this was a surveillance and diagnosis study of pathogens affecting domestic animals, IRB approval was waived. The sample collection was carried out by certified veterinarian following international standards for animal welfare. Guinea pig owners provided their consent for sampling and were informed of the study's outcome.

## 3 Results

### 3.1 Prevalence of protozoan and helminth parasites in guinea pig farms from Ecuador

There were 658 guinea pigs out of the 765 included in the study that carried at least a single parasite. This translates to an overall prevalence of parasitism of 86.0% (95% CI: 83.4–88.3). In Table 1, the prevalence of each genus of protozoan and helminth parasites is detailed.

**Table 1:** Prevalence of protozoan and helminth parasites in guinea pigs included in this study.

**Species**	**Positive guinea pigs**			**Parasite features**
	***N***	**Prevalence (%)**	**95% CI**	
*Balantidium* spp.	41	5.4%	4.0–7.2	Zoonotic
*Cryptosporidium* spp.	41	5.4%	4.0–7.2	Zoonotic
*Eimeria* spp.	387	50.6%	47.0–54.1	Zoonotic
*Entamoeba coli*	55	7.2%	5.6–9.2	Zoonotic
*Giardia* spp.	38	5.0%	3.6–6.7	Zoonotic
**Overall protozoan**	430	56.2%	52.7–59.7	
*Capillaria* spp.	37	4.8%	3.5–6.6	Panzootic
*Fasciola hepatica*	82	10.7%	8.7–13.1	Zoonotic
*Heterakis* spp.	14	1.8%	1.1–3.0	Panzootic (avian)
*Paraspidodera uncinata*	284	37.1%	33.8–40.6	Panzootic (rodents)
*Passalurus* spp.	82	10.7%	8.7–13.1	Panzootic (rodents)
*Trichostrongylus* spp.	240	31.4%	28.2–34.7	Zoonotic
*Trichuris* spp.	103	13.5%	11.2–16.1	Zoonotic
**Overall helminths**	536	70.1%	66.7–73.2	

Five different genera of protozoan parasites were found, including zoonotic species such as *Giardia, Cryptosporidium, Entamoeba, Eimeria*, and *Balantidium*. The overall prevalence of protozoan parasites was 56.21% (95% CI: 52.7–59.7). The most prevalent protozoan was *Eimeria*, with a value of 50.6% (95% CI: 47.0–54.1).

Seven different genera of helminth parasites (three of which are zoonotic) were found, including *Capillaria, Fasciola, Paraspidodera, Passalurus, Trichostrongylus, Trichuris*, and *Heterakis*. The overall prevalence of helminths was 70.1% (95% CI: 66.8–73.2). The most prevalent helminths were *Paraspidodera and Trichostrongylus*, with prevalence rates of 37.1% (95% CI: 33.7–40.6) and 31.4% (95% CI: 28.2–34.7), respectively.

In Table 2, the prevalence of multiple infections with two or more parasites is detailed. Co-infections with up to 7 different parasites in a single guinea pig were found. There were 241 guinea pigs infected with two parasites (31.5%), 154 with three parasites (20.1%), and 50 with four parasites (6.5%).

**Table 2:** Prevalence of co-infections with multiple parasites in the guinea pigs included in the study.

**Number of parasites**	**Number of guinea pigs**	**Prevalence (%)**	**95% CI**
7	1	0.1%	0–0.7
6	1	0.1%	0–0.7
5	8	1.1%	0.5–2.1
4	50	6.5%	5–8.5
3	154	20.1%	17.4–23.1
2	241	31.5%	28.3–34.9

### 3.2 Risk factor analysis for the prevalence of parasites in guinea pig farms from Ecuador

In Table 3, the Odds Ratios and *p*-values for the univariate and multivariate analysis for the several risk factors included in the study are detailed. Also, the prevalence of parasitism for each category within each risk factor is also included.

**Table 3:** Risk factor analysis for parasitism in guinea pigs.

**Risk factor**		**Parasitism (+)**	**Parasitism (–)**	**Prevalence (%)**	**Bivariate analysis**		**Multivariate analysis**	
					**OR (CI 95%)**	***p*** **-value**	**OR (CI 95%)**	***p*** **-value**
Type of farm	Backyard production	120	30	80.00%			1.01 (0.63–1.64)	0.93
	Small scale	504	77	86.75%	0.61 (0.38–0.97)	0.05		
	Large scale	34	0	100.00%	Undefined^*^	0.009		
Type of cage	Jaula	242	14	94.53%	3.86 (2.15–6.92)	0.0000025	0.28 (0.15–0.52)	**0.0001**
	Poza	416	93	81.73%				
Type of feeding	Forage	117	3	97.50%	7.49 (2.33–24.03)	0.0001	0.17 (0.05–0.56)	**0.0037**
	Forage + balanced feed	541	104	83.88%				
Contact with other animal species	Present	115	12	90.55%	1.67 (0.89–3.15)	0.14	2.11 (1.10–4.03)	**0.02**
	Absent	543	95	85.11%				
Vet management advisory	Present	56	0	100%	Undefined^*^	0.003	Undefined^*^	0.96
	Absent	602	107	84.91%				

Odd ratios (OR) for bi and multivariate analysis are detailed (CI: confidence interval).

^*^Undefined: Odd ratio not calculated due to zero cases.

Bold values are *p*-values statistically significant.

For the prevalence of parasitism in guinea pigs on the three different types of farms the values obtained were 80% (95% CI: 72.7–86.1), 86.75% (95% CI: 83.7–89.2), and 100% (95% CI: 89.7–100.0) for “backyard production”, “small scale farms”, and “large scale farms”, respectively. Although those differences were statistically significant in the univariate analysis, the number of guinea pigs was not a risk factor for parasitism in the multivariate analysis (*p* = 0.93).

For the prevalence of parasitism in guinea pigs for the two different types of cages, the values found were 81.73% (95% CI: 78.1–84.8) and 94.53% (95% CI: 90.9–96.9) for “poza” and “jaula”, respectively. Those differences were statistically significant either in the univariate of multivariate analysis (*p* < 0.001).

For the prevalence of parasitism in guinea pigs depending on the two different types of feeding, the values found were 97.5% (95% CI: 92.8–99.4) and 83.88% (95% CI: 80.8–86.5) for “fresh forage” and “mixture of fresh forage and balanced feed”, respectively. Those differences were statistically significant either in the univariate of multivariate analysis (*p* < 0.01).

For the prevalence of parasitism in guinea pigs considering the contact with other domestic animal species (present/absent, the values obtained were 90.55% (95% CI: 84.1–95.1) and 85.11% (95% CI: 82.1–87.6) for “present” and “absent”, respectively. While those differences were not statistically significant in the univariate analysis (*p* = 0.14), they reached the statistical significance in the multivariate analysis (*p* = 0.02).

Regarding the prevalence of parasitism in guinea pigs according to veterinarian management advisory (present/absent), the values found were 100% (95% CI: 93.6–100.0) and 84.91% (95% CI: 82.1–87.3) for “present” and “absent”, respectively. Although those differences were statistically significant in the univariate analysis, the number of guinea pigs was not a risk factor for parasitism in the multivariate analysis (*p* = 0.96).

## 4 Discussion

In the present study, a remarkable prevalence of 86% for enteric parasites in guinea pig farms from Ecuador was found, including five genera of protozoa and seven of helminths already described as zoonotic parasites in domestic animals. Moreover, mixed infections with two or more parasites were also persistent in guinea pigs. While the most prevalent protozoan parasite was *Eimeria*, with a prevalence of over 50%, other important zoonotic protozoans, such as *Giardia* and *Cryptosporidium*, were also found. *Three* zoonotic helminths were also found at prevalences over 10%: *Fasciola hepatica, Trichostrongylus*, and *Trichuris*. Overall, our results align with previous studies in Ecuador, Peru, Cameroon, and Benin, where the carriage of enteric parasites in livestock guinea pigs has already been reported (5, 6, 14–16). Moreover, various studies in Ecuador have shown that gastrointestinal parasitosis is a problem shared by different animal species, which allows the findings in guinea pigs to be put into context. For instance, the prevalence of helminths reached 27.4% and 74.32 in free roaming dogs from rural and urban settings in Ecuador, respectively (23, 24). In cattle, high infections rates of 87.3% and 31% for protozoa and helminths (31.0%) have been reported in Chimborazo province (14); an also an overall high parasitism rate of 82.44% have been reported in sheep (25), and 48.65% in backyard pigs (26). Our findings in guinea pigs support these pervious reports underscoring a widespread presence on enteric parasites across different species of domestic animals in Ecuador.

To the best of our knowledge, our study is the first report of *F. hepatica* in guinea pigs from Ecuador. In fact, there is only a single report from 1996 where *F. hepatica* was described in guinea pigs from Peru (27). This zoonotic parasite causes fasciolosis, a neglected disease in South America, which is associated with severe hepatic disease (28, 29). Human fasciolosis outbreaks have been reported in Ecuador (28), and animal reservoirs, including cattle, sheep, and pigs, have been previously identified (29). Our results support that guinea pigs may also play a role as reservoirs of *F. hepatica* in in the Andean region.

The zoonotic parasites found in our research have substantial implications for public health. For instance, *Eimeria, Giardia*, and *Cryptosporidium* are well-known agents that cause diarrhea, and helminths such as Trichuris also cause gastrointestinal problems (6, 18, 30). In general, these enteric parasites are involved in the burden of disease in rural settings from low- and middle-income countries like Ecuador, including child malnutrition and stunting (18). Moreover, cryptosporidiosis as a foodborne zoonosis of veterinary and public health concern more associated to small livestock like guinea pigs (30). In this sense, guinea pig backyard production was analyzed as one of the categories in the variable type of farm. Although backyard production was not found to be a risk factor for parasitism, the high level of parasites prevalence represents a public health threat. In the context of the Andean region, this guinea pig backyard production includes a very traditional breeding in indigenous communities where the animals grow in the kitchen area within the household in a very close interaction with food, kitchen stuff and humans itself. Public policies dedicated to rise awareness in managing parasitic infections would help to prevent transmission to humans, as it has been suggested for other domestic animals in close contact with humans like horses (31).

The impact of basic guinea pig breeding parameters on the level of enteric parasitism in guinea pigs was assessed in this study to the best of our knowledge. In these sense, three risk factors were found in the multivariate analysis. First, the type of cage used for guinea pig breeding had an impact on the prevalence of parasitism, with “poza” having a smaller prevalence compared to “jaula”. Although the accumulation of fecal residues (meaning a higher risk for parasite exposure) is expected to be larger over the dirt ground in “poza” than in “jaula”, the results could be explained by the fact that the “poza” is cleaned more frequently compared to the “jaula” (reported by producers to the authors). Second, feeding guinea pigs exclusively on fresh forage was also associated with a higher prevalence of parasitism in guinea pigs compared to balanced feed supplementation, which may be linked to a better immune status link to the balance feeding; but also to the fact that fresh forage came from pastures also use to feed cattle and contamination with fecal material (and parasites) happened. Third, the prevalence of parasite infection was higher in guinea pigs exposed to other domestic animals like dogs, cats, pigs or cattle, underscoring transmission of parasites across domestic species due to the panzootic nature of these pathogens. Nevertheless, we draw attention to the prevalence of parasitism, which was generally high. Still, the differences in risk factors analyzed indicate that sanitary interventions would have an impact that warrants further research and standardization. In this sense, other control measures and ethnoveterinary approaches could also be considered in guinea pig farming, as its has already been reported for sustainable parasite control in small livestock like poultry (32, 33).

Our study has some limitations that we would like to acknowledge. First, as a convenience sampling was used, potential bias cannot be totally rule out, and either overall parasitic prevalence values and risk factor analysis should be taken with caution; further studies involving larger animal samples and farms for more provinces of Ecuador should follow. Second, our diagnosis was based in traditional morphological identification under the microscope without confirmation by molecular methods; further studies should include molecular diagnosis to improve the sensitivity and specificity of the parasites species identified, as it has already been done in small ruminants (34, 35).

In conclusion, our study highlights the guinea pig as a reservoir for zoonotic enteric parasites in Ecuador. Those findings underscore the need for further research with a One Health perspective to enhance livestock guinea pig health and productivity. This field of study is fundamental to developing effective practice guidelines for guinea pig farming, which will enhance production, mitigate occupational risks for breeders, and reduce foodborne transmission. This is particularly relevant in the context of rural Andean communities, where guinea pigs are part of the diet for millions of people, often in underserved communities, where food security is crucial in the fight against child malnutrition.

## Data Availability

The original contributions presented in the study are included in the article/Supplementary material, further inquiries can be directed to the corresponding author.
