# Supplementary material for: Livestock guinea pigs in Ecuador as reservoirs of zoonotic protozoa and helminths
Source: Front Vet Sci. 2025 Sep 24;12:1658485. doi: 10.3389/fvets.2025.1658485 (PMC12504026; doi:10.3389/fvets.2025.1658485)
Supplement: Supplementary file 1 [file Table_1.docx]

**Supplementary Table 1. Variables and categories analyzed for the guinea pig farms included in the study.**

| **Variable (Risk factor)** | **Categories** |
| --- | --- |
| Number of animals per farm | 1. Backyard production (<100 animals)  2. Small scale farm (101-500 animals)  3. Large scale farm (>500 animals) |
| Type of cage | 1. Jaula (suspended wire cage suspended)  2. Poza (wooden fence on a dirt ground) |
| Type of feeding | 1. Fresh forage  2. mixed fresh forage/balanced feed |
| Other domestic animals in the farm | 1. Present  2. Absent |
| Veterinary care counseling | 1. Present  2. Absent |
